# Association of whole blood copper, magnesium and zinc levels with metabolic syndrome components in 6–12-year-old rural Chinese children: 2010–2012 China National Nutrition and Health Survey

**DOI:** 10.1186/s12986-021-00593-w

**Published:** 2021-06-27

**Authors:** Huidi Zhang, Qingqing Man, Pengkun Song, Siran Li, Xiaobing Liu, Lijuan Wang, Yuqian Li, Yichun Hu, Lichen Yang

**Affiliations:** National Institute for Nutrition and Health, Chinese Center for Disease Control and Prevention, Key Laboratory of Trace Element Nutrition, National Health Commission of the People’s Republic of China, Beijing, People’s Republic of China

**Keywords:** MetS components, Whole blood, Copper, Zinc, Magnesium, Chinese children

## Abstract

**Background:**

Metabolic syndrome (MetS) is significantly associated with the risk of cardiovascular disease and its prevalence is showing a trend of getting younger. Previous studies on the relationship between elements and MetS were mostly reported in adults with single element analysis, while reports in children with combined effects of multiple elements were very limited. The aim of this study is to investigate the association between whole blood Cu, Mg and Zn in both single and combined effects and MetS components in rural Chinese children aged 6–12 years based on the data from 2010–2012 China National Nutrition and Health Survey.

**Methods:**

A total of 911 children (51.2% male, 48.7% female) aged 6–12 years were included. Basic characteristics and MetS component parameters were collected and determined by trained stuffs. Elements were detected by Inductively Coupled Plasma Mass Spectrometry (ICP-MS). Multivariate logistic regression analysis was performed to examine the independent relationship between elements and MetS components.

**Results:**

In single metal analysis, copper was positively associated with elevated waist (OR = 2.00, 1.18–3.28) and all of the metals were associated with elevated TG. And the comprehensive analysis of multiple elements were mostly consistent with the results of single element analysis (low Cu + high Zn with elevated TG (OR = 2.21, 1.18–4.13), high Cu + low Mg with elevated TG (OR = 0.40, 0.16–0.95), high Cu + high Mg with elevated waist (OR = 2.03, 1.26–3.27)), except the combination of Zn and Mg (high Zn + low Mg with reduced HDL-C (OR = 0.47, 0.28–0.77)).

**Conclusions:**

Our study suggested Cu, Zn and Mg in children are indeed associated with metabolic syndrome components, whether in single element or multi-element combined analysis. The results will be confirmed through additional cohort research.

## Introduction

Metabolic syndrome (MetS) is associated with a cluster of related risk factors of metabolic origin that accelerates the development of cardiovascular disease and increase the risk for development of type 2 diabetes [1]. The prevalence of the chronic disease is increasing rapidly and showing a tendency to affect the younger people. According to the report of 2010–2012 China National Nutrition and Health Survey, the prevalence of metabolic syndrome in urban and rural children aged 10-17y were 4.7% and 3.9%, respectively. 35.7% of children aged 7-9y had at least one abnormal component of metabolic syndrome, and the proportion in rural areas was higher than that in urban areas (37.3% vs. 34.0%) [2, 3].

Several studies [4–6] have reported the relationship between Cu, Zn, Mg and MetS, but most of them are limited to adults and only focused on one element. The combined effects of multi elements in children are rarely reported. The Dongfeng cohort in China firstly explored the relationship between 23 elements in the plasma of the elderly and type 2 diabetes [7], and found that the combined effect of multi-element is different from that of single element analysis.

In this cross-sectional study, we aim to investigate the association between single and combined effect of whole blood Cu, Mg, Zn and MetS components in Chinese children aged 6–12 years from the 2010–2012 China National Nutrition and Health Survey (CNNS).

## Materials and methods

### Subjects

The study was nested in the 2010–2012 CNNS, which was a cross-sectional, nationally-representative survey. According to the estimation of sample size based on population rate [8], the formula is as following:

$${\text{N}} = {\text{Z}}_{{\alpha /{\text{2}}}} ^{{\text{2}}} /\delta ^{{\text{2}}} *\pi *({\text{1}} - \pi )$$

with 95% confidence interval, α = 0.05, Z_α/2_ = 1.96; the allowable error δ = π* r, π is the expected prevalence rate, the relative error r is 10–20%

As the prevalence rate of 7–9 years old children in rural China who had at least one of the four components of MetS is 37.3% [3]. The minimum sample size is 162.

According to the regional type and geographical distribution of different monitoring points, the children were sampled by multi-stage stratified random sampling method through PROC SURVEY commands in SAS 9.4. 911 children aged 6–12 years (half female and half male) were finally recruited from 72 rural areas in this study.

All children and their guardians gave informed consent for inclusion before they participated in the study. The study protocol was approved by the Ethics Committee of the Institute for Nutrition and Food Safety, Chinese Center for Disease Control and Prevention (now known as the National Institute for Nutrition and Health, China CDC).

### Data collection

Physical examinations were performed by trained medical staff following standardized procedures. Height was measured by a Seca 213 Portable Stadiometer Height-Rod with a precision of 0.1 cm. Body weight was measured by a Seca 877 electronic flat scale with a precision of 0.1 kg. During the measurements, each child was required to take off shoes, hat, and coat, and females untied their braids to ensure the accuracy of measurements. Body mass index (BMI) was calculated as weight (kg)/square of height (m^2^). Waist circumference was measured two centimeters above the navel by a tape with a precision of 0.1 cm. Systolic blood pressure (SBP, mmHg) and diastolic blood pressure (DBP, mmHg) were measured in the right upper arm using a mercury sphygmomanometer. The blood pressure of each child was recorded as the average of three measurements. Venous blood was collected from all children in the morning after at least 10 h of fasting, and each blood sample was divided into an anticoagulation tube and serum separator tube. Blood samples in serum separator tubes were promptly centrifuged at 3000 g for 15 min, 20–30 min after the blood was taken and then divided into serum aliquots and frozen at − 80 °C for subsequent assays. Serum fasting glucose (FG), high density lipoprotein-cholesterol (HDL-C), and triglyceride (TG) levels were measured by an enzymatic method using a Hitachi 7600 automatic biochemical analyzer (Japan). Whole blood Cu, Mg and Zn concentrations were measured by inductively coupled plasma mass spectrometry (NexION 350D, PerkinElmer) from the anticoagulation tube in KineticEnergy Discrimination (KED) model. The accuracy of the method was measured with certified reference material (Seronorm TM Trace Elements Whole Blood L-2, Norway). The limit of detection of the method of Zn, Mg and Cu were: 0.22 μg/L, 1.02 μg/L and 2.49 μg/L, respectively. The recovery was as follows: 110.1% (Zn), 100.8% (Cu) and 100.1% (Mg).

### Definition of MetS and its components

Since there was no acknowledged standard for judging MetS in children aged 6–10 years, we adopted a modified criteria for MetS recommended by the American Academy of Pediatrics and International Diabetes Federation (AAP & IDF) [9]. The MetS components were defined as follows: (1) obesity: waist circumference ≥ 95th percentile of children of the same age and sex, or BMI ≥ 95th percentile of children of the same age and sex; (2) hypertension: blood pressure ≥ 95th percentile of children of the same age and sex (fast identification: systolic BP ≥ 120 mmHg or diastolic BP ≥ 80 mmHg); (3) dyslipidemia: Reduced HDL-C (< 1.03 mmol/L); or Elevated TG (≥ 1.47 mmol/L); (4) hyperglycemia: FG ≥ 5.6 mmol/L.

### Statistic analysis

Statistical analyses were performed using SPSS version 19.0. The results of descriptive characteristics were expressed as average ± standard deviation. The relationships among clinical indexes and the number of MetS components were analyzed using the Kruskal–Wallis test. Spearman correlation coefficients were applied to assess the relationship between metals and MetS components. The odds ratios (ORs) and 95% confidence intervals (95%CIs) were determined by multivariate logistic regression to investigate the associations between MetS components and tertiles of whole blood metals. We categorized the level of whole blood Cu, Zn, the ratio of Cu to Zn, and Mg into tertiles and used the lowest tertile as the reference. P-trend analysis involved treating the tertiles as a continuous variable in regression analyses. The combination of metals defined as low was a concentration < 50% of the range, and those defined as high was the concentration ≥ 50% percentage whole value. All statistical tests were two-sided and statistical significance was considered at *P* < 0.05.

## Results

### Characteristics of the study population

911 individuals (444 females, 467 males) with complete data on MetS components and whole blood Cu, Mg and Zn concentrations were included in this study. Basic characteristics of the children are shown in Table 1. There were sex-based differences in the comparison of clinical and biological indexes: the waist circumference, HDL-C and Cu concentrations, and Cu/Zn ratio were higher in males, but the TG concentration was higher in females. We also analyzed these variables using different numbers of MetS components. There was 45.4% of the participants who had at least one metabolic syndrome component abnormal. We observed that except HDL-C, other significant indexes all exhibited an ascending trend with an increasing number of MetS components.

**Table 1:** Basic characteristics of children according to sex and the number of MetS components

Indexes	Total (N = 911)	Male (N = 467)	Female (N = 444)	MetS components		
				0(N = 497)	1(N = 336)	≥ 2(N = 78)
Age(years)	9.12 ± 1.70	9.01 ± 1.70	9.23 ± 1.70	8.93 ± 1.69	9.31 ± 1.68	9.51 ± 1.68^#^
Height (m)	131.52 ± 11.84	131.06 ± 11.41	132.01 ± 12.27	130.08 ± 10.71	132.71 ± 12.58	135.59 ± 13.87^#^
Weight (kg)	28.95 ± 8.74	29.17 ± 8.96	28.71 ± 8.49	26.95 ± 6.81	30.33 ± 9.27	35.74 ± 12.21^#^
Waist (cm)	55.96 ± 7.91	56.76 ± 7.93	55.11 ± 7.82*	53.40 ± 5.01	57.58 ± 8.69	65.25 ± 10.41^#^
BMI (kg/m^2^)	16.41 ± 2.78	16.66 ± 2.98	16.15 ± 2.53	15.72 ± 2.27	16.86 ± 2.82	18.92 ± 3.65^#^
Obesity (%)	16.02%	14.56%	17.56%	0%	27.08%	70.51%^#^
TG (mmol/L)	0.79 ± 0.48	0.74 ± 0.46	0.85 ± 0.50*	0.67 ± 0.29	0.93 ± 0.62	1.00 ± 0.56^#^
HDL-C (mmol/L)	1.27 ± 0.30	1.31 ± 0.30	1.23 ± 0.29*	1.40 ± 0.26	1.15 ± 0.28	1.01 ± 0.21^#^
FG (mmol/L)	4.85 ± 0.71	4.9 ± 0.73	4.8 ± 0.68	4.71 ± 0.53	4.94 ± 0.77	5.36 ± 1.08^#^
SBP(mmHg)	92.44 ± 11.42	92.68 ± 10.88	92.19 ± 11.96	91.44 ± 10.16	92.92 ± 12.31	96.77 ± 13.73^#^
DBP (mmHg)	60.23 ± 8.65	60.23 ± 8.07	60.23 ± 9.24	59.41 ± 7.72	60.77 ± 9.47	63.18 ± 9.77^#^
Cu (mg/L)	1.01 ± 0.13	1.03 ± 0.14	0.99 ± 0.13*	1.00 ± 0.13	1.01 ± 0.13	1.07 ± 0.14^#^
Zn (mg/L)	5.21 ± 1.07	5.19 ± 1.06	5.24 ± 1.08	5.21 ± 1.05	5.22 ± 1.09	5.19 ± 1.11
Cu/Zn	0.20 ± 0.05	0.20 ± 0.05	0.20 ± 0.04*	0.20 ± 0.04	0.20 ± 0.05	0.21 ± 0.04^#^
Mg (mg/L)	41.44 ± 5.28	41.29 ± 5.24	41.6 ± 5.32	41.16 ± 5.26	41.74 ± 5.34	41.92 ± 5.04

BMI, body mass index; TG, triglycerides;HDL-C, high density lipoprotein-cholesterol; FG, fasting glucose;SBP, systolic blood pressure;DBP, diastolic blood pressure; *p < 0.05 for sex; #*p* < 0.05 for trend

### The association between tertiles of Cu, Zn, Cu/Zn and Mg and MetS component indicators

The relationships between Cu, Zn, Cu/Zn and Mg and MetS component indicators are shown in Table 2. The values of BMI, DBP and waist circumference tended to be higher in the upper tertiles of Cu, whereas the TG value was lowest in the third tertile of Cu. For blood Zn, both DBP and SBP had an ascending trend with increasing tertiles of Zn levels. But in the Cu/Zn group, the trend was opposite, with the values of DBP, SBP and TG decreased as Cu/Zn tertiles increased. In the Mg group, there was a significantly ascending trend for DBP, FG and TG values as the Mg tertiles increased. Whole blood Cu was positively associated with DBP and waist circumference, and negatively associated with TG. There were positive correlations between Zn and FG, TG, DBP and SBP. In contrast to the Cu correlations, the ratio of Cu to Zn was negatively correlated to DBP, SBP and TG. However, waist circumference was also positively associated with the Cu/Zn ratio. Blood Mg levels showed a correlation only with DBP.

**Table 2:** Distribution of metabolic syndrome component indicators by tertiles (T1–T3) of blood copper (Cu), zinc (Zn), the Cu/Zn ratio and ³ã³ (Mg) levels

Indexes	Cu (mg/L)				Zn (mg/L)				Cu/Zn			Mg (mg/L)				
	T1 < 0.94	T2 0.94- 1.06	T3 > 1.06	*P value*	T1 < 4.64	T2 4.64- 5.70	T3 > 5.70	*P value*	T1 < 0.17	T2 0.17- 0.21	T3 > 0.21	*P value*	T1 < 38.89	T2 38.89–43.66	T3 > 43.66	*P value*
N = 911	294	315	302		300	310	301		295	311	305		300	310	301	
BMI (kg/m^2^)	16.16	16.23	16.85	**0.004**	16.21	16.45	16.58	0.250	16.45	16.48	16.31	0.733	16.25	16.56	16.42	0.388
Waist (cm)	55.91	55.21	56.78^*^	**0.046**	55.52	55.98	56.36	0.430	55.96	56.36	55.54^*^	0.444	55.13	56.10	56.64	0.060
TG (mmol/L)	0.86	0.78	0.74^#^	**0.011**	0.74	0.82	0.82^*^	0.063	0.91	0.75	0.72^#^	**0.001**	0.73	0.85	0.79	**0.010**
HDL-C (mmol/L)	1.28	1.27	1.26	0.085	1.25	1.28	1.28	0.325	1.28	1.28	1.26	0.695	1.26	1.28	1.27	0.735
FG (mmol/L)	4.79	4.89	4.88	0.201	4.82	4.81	4.93^*^	0.062	4.87	4.87	4.82	0.601	4.81	4.80	4.95	**0.015**
SBP (mmHg)	92.27	91.94	93.13	0.412	90.54	92.94	93.83^*^	**0.001**	93.86	92.84	90.67^#^	**0.002**	91.22	92.84	93.25	0.070
DBP (mmHg)	59.28	60.21	61.19^*^	**0.026**	58.58	60.95	61.15^*^	**0.001**	61.17	60.35	59.21^#^	**0.020**	58.98	60.56	61.15^*^	**0.006**

The bold numbers,* P* value was < 0.05, and the difference was statistically significant

BMI, body mass index; DBP, diastolic blood pressure; FG, fasting glucose; HDL-C, high density lipoprotein-cholesterol; SBP, systolic blood pressure; TG, triglycerides; *positive correlation; ^#^negative correlation

### Odds ratios for MetS components in tertiles of Cu, Zn, Cu/Zn and Mg

Table 3 summarizes the ORs and 95%CIs for MetS components associated with concentrations of whole blood Cu, Mg and Zn, and the Cu/Zn ratio, each categorized into tertiles. For elevated waist circumference measurements, significant associations were found for Cu and Cu/Zn using a crude model. After adjusting for age, BMI and sex, the multivariable adjusted ORs (95%CIs) were 2.00 (1.56–3.89) for Cu and 2.08 (1.22–3.55) for Cu/Zn. No significant correlations were found between Mg or Zn and elevated waist. All of the metals were associated with elevated TG, either in the crude or adjusted model. The risk of elevated TG was significantly decreased in the tertile 3 group compared with the tertile 1 (T1) group for Cu and Cu/Zn values. The ORs and 95% CIs for Cu and Cu/Zn were 0.34 (0.17–0.66) and 0.31 (0.15–0.62), respectively, in the crude model. These trends persisted after adjustment, with Cu and Cu/Zn showing ORs (95%CI) of 0.33 (0.16–0.65) and 0.35 (0.17–0.71), respectively. However, TG levels were inversely associated with Mg and Zn. The ORs (95%CI) for elevated TG in individuals categorized in the Mg and Zn tertile 2 (T2) groups were 2.36 (1.20–4.62) and 2.27 (1.18–4.35), respectively, without adjustment. The ORs (95%CIs) were attenuated but remained statistically significant after adjustment [T2 vs. T1: 2.12 (1.10–4.10) for Zn, 2.34 (1.19–4.61) for Mg]. There was no significant association between metals and hypertension, reduced HDL-C and hyperglycemia.

**Table 3:** Odds ratios (95% confidence intervals) for metabolic syndrome components according to whole blood metals distribution

Index	Cu				Zn				Cu/Zn				Mg			
	T1	T2	T3	*P*	T1	T2	T3	*P*	T1	T2	T3	*P*	T1	T2	T3	*P*
N = 911	294	315	302		300	310	301		295	311	305		300	310	301	
Elevated Waist																
n	32	44	70		51	50	45		37	50	59		40	49	57	
Crude	1	1.32 (0.82–2.16)	**2.47** **(1.56–3.89)**	**0.001**	1	0.93 (0.61–1.43)	0.85 (0.55–1.32)	0.789	1	1.33 (0.84–2.11)	**1.67** **(1.07–2.61)**	**0.053**	1	1.22 (0.77–1.91)	1.51 (0.97–2.35)	0.172
Adjusted	1	1.23 (0.71–2.12)	**2.00** **(1.18–3.28)**	**0.001**	1	0.78 (0.48–1.28)	0.62 (0.37–1.04)	0.194	1	1.32 (0.77–2.28)	**2.08** **(1.22–3.55)**	**0.019**	1	1.21 (0.71–2.04)	1.58 (0.95–2.62)	0.200
Hypertension																
n	9	7	13		7	8	14		14	10	5		9	9	11	
Crude	1	0.72 (0.26–1.95)	1.42 (0.59–3.38)	0.334	1	1.10 (0.39–3.09)	2.04 (0.81–5.13)	0.205	1	0.66 (0.29–1.52)	0.33 (0.12–0.94)	0.096	1	0.96 (0.37–2.47)	1.22 (0.50–3.00)	0.849
Adjusted	1	0.94 (0.33–2.64)	1.95 (0.79–4.82)	0.175	1	0.91 (0.32–2.60)	1.48 (0.70–2.85)	0.513	1	0.83 (0.35–1.93)	0.58 (0.20–1.69)	0.610	1	0.85 (0.32–2.24)	1.04 (0.42–2.61)	0.909
Elevated TG																
n	34	20	13		14	31	22		34	21	12		13	30	24	
Crude	1	**0.51** **(0.29–0.92)**	**0.34** **(0.17–0.66)**	**0.002**	1	**2.27** **(1.18–4.35)**	1.61 (0.80–3.21)	**0.041**	1	**0.55** **(0.31–0.93)**	**0.31** **(0.15–0.62)**	**0.002**	1	**2.36** **(1.20–4.62)**	1.91 (0.95–3.83)	**0.036**
Adjusted	1	**0.52** **(0.28–0.93)**	**0.33** **(0.16–0.65)**	**0.002**	1	**2.12** **(1.10–4.10)**	1.41 (0.70–2.85)	0.066	1	0.57 (0.32–1.02)	**0.35** **(0.17–0.71)**	**0.010**	1	**2.34** **(1.19–4.61**)	1.81 (0.90–3.66)	**0.048**
Reduced HDL-C																
n	62	62	75		74	66	59		53	73	73		68	64	67	
Crude	1	0.91 (0.61–1.36)	1.23 (0.84–1.81)	0.281	1	0.82 (0.56–1.20)	0.74 (0.51–1.09)	0.310	1	1.40 (0.94–2.08)	1.43 (0.96–2.13)	0.145	1	0.88 (0.60–1.30)	0.97 (0.66–1.43)	0.815
Adjusted	1	0.90 (0.61–1.35)	1.23 (0.83–1.82)	0.282	1	0.81 (0.55–1.18)	0.72 (0.49–1.07)	0.266	1	1.40 (0.93–2.09)	1.48 (0.98–2.23)	0.132	1	0.88 (0.60–1.31)	0.97 (0.66–1.42)	0.828
Hyperglycemia																
n	25	37	32		37	26	31		25	33	36		31	25	38	
Crude	1	1.43 (0.83–2.44)	1.27 (0.73–2.21)	0.414	1	0.65 (0.38–1.10)	0.81 (0.49–1.35)	0.277	1	1.28 (0.74–2.13)	1.44 (0.84–2.47)	0.399	1	0.76 (0.43–1.32)	1.25 (0.75–2.07)	0.180
Adjusted	1	1.57 (0.91–2.70)	1.36 (0.77–2.39)	0.429	1	0.62 (0.36–1.06)	0.75 (0.45–1.26)	0.212	1	1.40 (0.80–2.44)	1.69 (0.97–2.95)	0.177	1	0.73 (0.41–1.27)	1.22 (0.73–2.03)	0.108

The bold numbers,* P* value was < 0.05, and the difference was statistically significant

Adjusted = adjusted for age, BMI, sex; Crude = non-adjusted; HDL-C, high density lipoprotein-cholesterol; *P, P* for trend; TG, triglycerides; T, tertile

### Odds ratios for MetS components associated with tertiles of the combination of metals

**Fig. 1:**
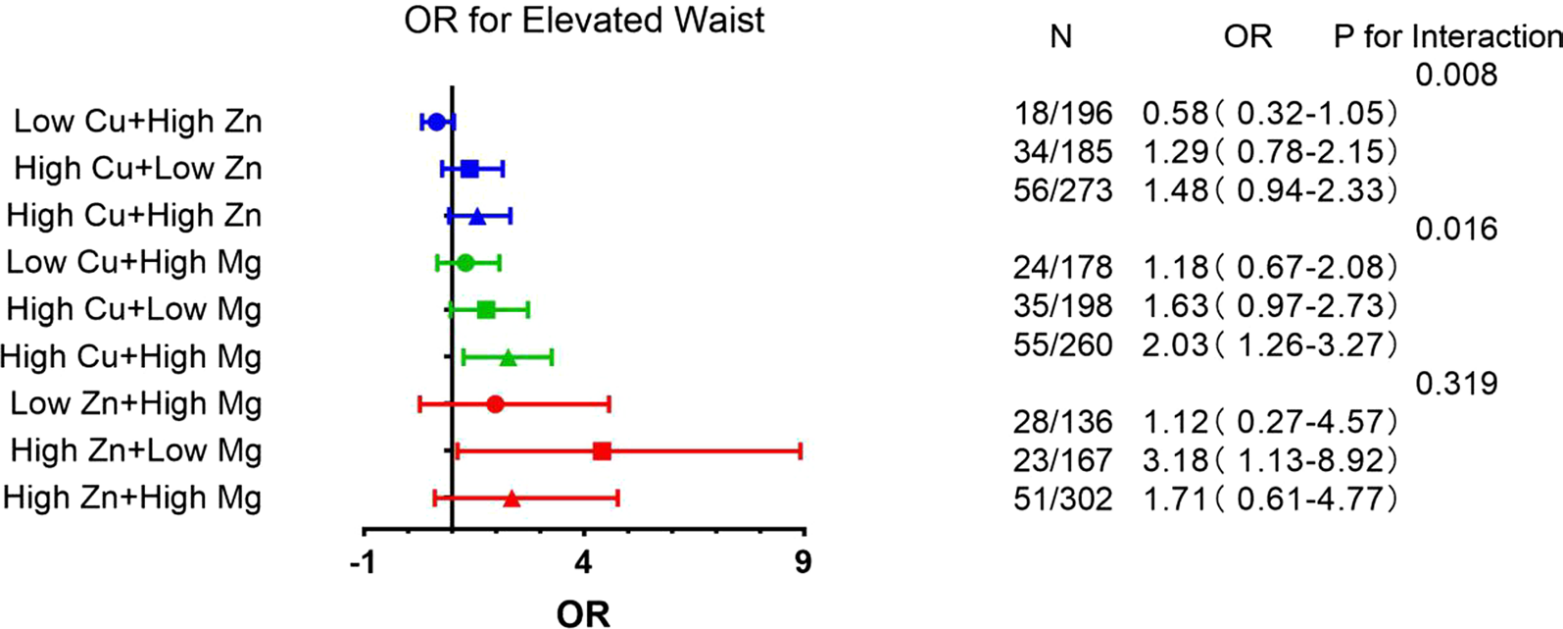
Associations of blood element levels with elevated waist. HDL-C, high density lipoprotein-cholesterol; TG, triglycerides; Low means < 50% concentration; High means ≥ 50% concentration; Low Cu + Low Zn, Low Cu + Low Mg and Low Zn + Low Mg were taken as the reference group; Shown are the odds ratios (ORs) and 95% confidence intervals in parentheses

**Fig. 2:**
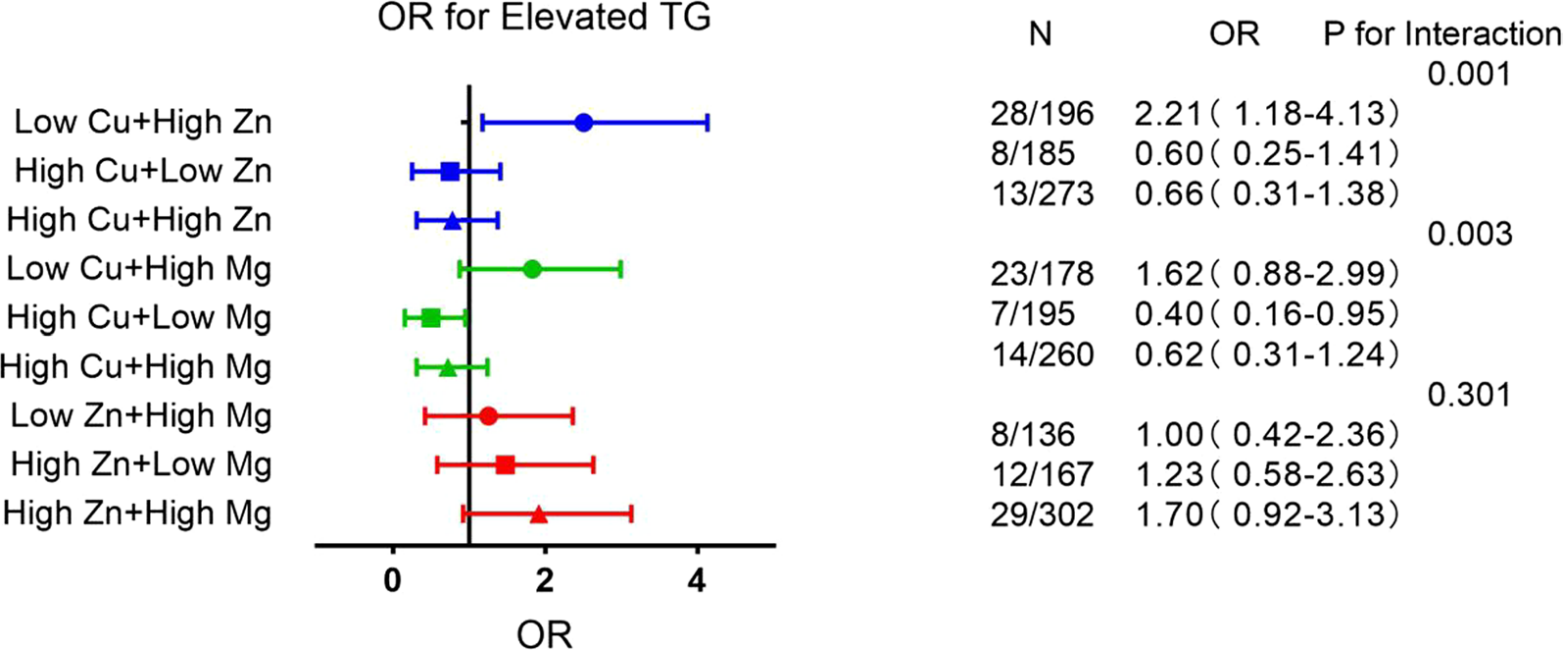
Associations of blood element levels with elevated triglycerides. HDL-C, high density lipoprotein-cholesterol; TG, triglycerides; Low means < 50% concentration; High means ≥ 50% concentration;Low Cu + Low Zn, Low Cu + Low Mg and Low Zn + Low Mg were taken as the reference group; Shown are the odds ratios (ORs) and 95% confidence intervals in parentheses

**Fig. 3:**
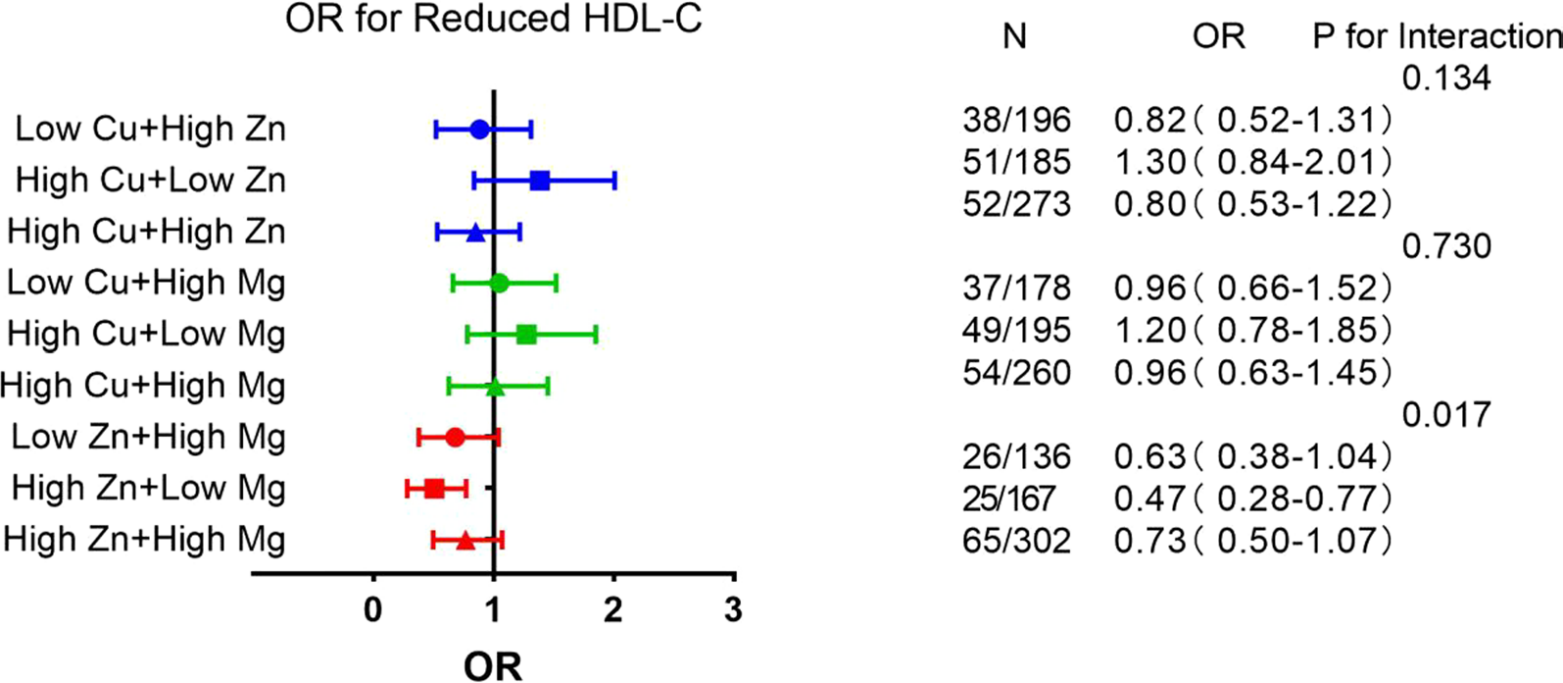
Associations of blood element levels with reduced HDL-C. HDL-C, high density lipoprotein-cholesterol; TG, triglycerides; Low means < 50% concentration; High means ≥ 50% concentration; Low Cu + Low Zn, Low Cu + Low Mg and Low Zn + Low Mg were taken as the reference group; Shown are the odds ratios (ORs) and 95% confidence intervals in parentheses

As the elements were all associated with MetS components, we combined each of them in pairs (Cu with Zn, Cu with Mg, Zn with Mg) to investigate joint correlations. In the joint analysis of Cu and Zn, the combination of low Cu and high Zn increased the risk of elevated TG [OR (95%CI), 2.21 (1.18–4.13)], when compared with the joint analysis of low Cu and low Zn. There was no significant association between MetS components and other Cu and Zn combinations. Correlations were also observed when we considered the interaction of Cu and Mg with MetS components. High Cu and high Mg were associated with an increased risk of elevated waist circumference [OR (95%CI), 2.03 (1.26–3.27)]. But when high Cu was combined with low Mg, the risk of elevated TG was attenuated to 0.40 (0.16–0.95). In the joint analysis of Zn and Mg, a significant correlation was only found for reduced HDL-C. We observed a negative association between reduced HDL-C and high Zn with low Mg. There was no association between the combination of Mg and Zn and other MetS components. The statistically significant results were shown in Figs. 1, 2 and 3. And we also summarized the meaningful results of single and combined analysis in Table 4.

**Table 4:** Summary of the significant results of the odds ratios for MetS components associated with single and combined elements

Single	Cu	Zn	Mg	Cu/Zn
	T3	T2	T2	T3
Elevated waist	**2.00 (1.18–3.28)**	**–**	**–**	**2.08 (1.22–3.55)**
Elevated TG	**0.33 (0.16–0.65)**	**2.12 (1.10–4.10)**	**2.34 (1.19–4.61)**	**0.35 (0.17–0.71)**
Reduced HDL-C	**–**	**–**	**–**	**–**

Combined	Cu + Zn	Cu + Mg		Mg + Zn
	Low Cu + High Zn	High Cu + Low Mg	High Cu + High Mg	High Zn + Low Mg
Elevated waist	**–**	**–**	**2.03 (1.26–3.27)**	**–**
Elevated TG	**2.21 (1.18–4.13)**	**0.40 (0.16–0.95)**	**–**	**–**
Reduced HDL-C	**–**	**–**	**–**	**0.47 (0.28–0.77)**

The bold numbers,* P* value was < 0.05, and the difference was statistically significant

HDL-C, high density lipoprotein-cholesterol; TG, triglycerides;Low means < 50% concentration; High means ≥ 50% concentration; Low Cu + Low Zn, Low Cu + Low Mg and Low Zn + Low Mg were taken as the reference; Shown are the odds ratios (ORs) and 95% confidence intervals.; -, the results didn’t have statisticlly significance

## Discussion

In this study, we explored whether the level of metals would affect the risk of metabolic syndrome components in children. And we hope to provide new ideas for further understanding of the occurrence and development of chronic diseases. The present result shows that the whole blood Cu, Zn and Mg, either alone or in combination, have association with MetS components. There was almost half (45.4%) of the participants who had at least one metabolic syndrome component abnormal. Therefore, a more systematic analysis of the relationship between element levels and metabolic syndrome components in children is necessary and meaningful.

Cu, Zn and Mg are all important elements involved in various metabolic pathways [10, 11]. Cu acts as an electron transfer intermediate in redox reactions [12, 13], which has a high level of oxidation and may lead to excessive damaging reactive oxygen species (ROS) by redox reactions [14, 15]. Zn as a co-factor of antioxidant enzymes, also plays an important role in oxidative stress and inflammation [16–18]. As the second most abundant intracellular ion, the deficiency of Mg is associated with coronary heart disease and type 2 diabetes[19]. One hypothesis suggests that a key mechanism leading to MetS involves oxidative stress caused by redox imbalance [20]. Thus, these elements may be related to the development of MetS.

In our study, whole blood concentrations of Cu and Zn were all in the normal range (0.61–1.9 mg/L Cu, 3.1–9.8 mg/L Zn), referring to a study of Swedish adolescents [21]. We observed that a higher level of whole blood Cu leads to an increased risk of elevated waist, which was representative of obesity. Catherine et al. [22] observed a positive relationship between serum Cu and abdominal obesity. We also found that the risk of elevated TG was decreased with ascending tertiles of Cu. It is acknowledged that Cu affects lipid metabolism, but the reported effects of Cu on different index of lipid metabolism remains inconsistent. Some studies suggested that serum Cu levels were positively associated with total cholesterol, TG or low density lipoprotein, such as those reported in Kuwaiti people aged 15–80 yea rs [23] and young American adults [24]. However, there were no associations between serum Cu and TG levels among children and adolescents in the NHANES 2011–2014 [25]. Besides, Leslie et al. [26] reported that increased total cholesterol and reduced HDL-C levels were observed in children with low Cu levels, which was consistent with our results.

In the current study, we also observed that tertiles of Zn were positively associated with the risk of elevated TG, in agreement with studies of Tehran’s urban population (OR = 1.60) [27] and Korean adults (OR = 1.47) [28]. Some research indicated that Zn overload would lead to Cu deficiency and reduced HDL-C concentrations [29]. As a consequence, the interaction of Cu and Zn should not be mutually exclusive, as they act as residual confounding factors for each other. This suggests that we need to reconsider previous analysis of single elements and consider interactions among these metals. Sukalski et al. found that a reduced serum Cu concentration leads to reduced Cu/Zn superoxide dismutase activity [30]. The Cu/Zn ratio can be used to determine the level of oxidative stress. In this study, all the values of Cu/Zn were less than 1.0, indicating the antioxidant mechanism in this population was normal [31]. In addition, the correlations of Cu/Zn with elevated waist and elevated TG were consistent with the correlations found with Cu, and with the findings reported by Fedor et al. [32]. Actually, in our investigation of the combination of Cu and Zn, we found that there was a positive association of high Zn and low Cu with elevated TG, which was in accordance with single element analysis.

As for the result of Mg, unlike some reports indicating that Mg supplements reduced TG levels and blood pressure [33], we observed an increased risk of elevated TG with the second tertile of Mg. Other studies suggested that Mg supplements were negatively associated with the risk of MetS [34, 35], so it is possible that low levels of Mg may be associated with some MetS components. In the current study, the risk of reduced HDL-C was decreased by the combination of a high Zn level and a low Mg level. There was a significant association betweena high Cu level combined with a low Mg level and the risk of elevated TG, which was consistent with the function of single metals. When Cu and Mg were both at high levels, the risk of an elevated waist circumference was raised. This was in agreement with a cross-sectional study conducted by Beydoun et al. [36].

The current study has several strengths. Firstly, this study focused on comprehensively exploring the effects of elements on all of the metabolic syndrome components in children. It could provide new avenues for the early screening of chronic diseases and protect the health of children and even for the elderly. Secondly, this is the first article that uses data from nationally representative cross-sectional study, to assess the relationship between elements and MetS components in rural children. It can provide a more comprehensive and detailed supplement to reflect the affect of elements on chronic diseases on Chinese children. Thirdly, it took the preliminary exploration of the combined effect of multiple elements in MetS components, which could provide new ideas for further understanding of the occurrence and development of disease.

There are also some limitations in this study. Firstly, there was no acknowledged ideal biomarker for the nutrition status of Cu, Mg, Zn, we adopted the whole blood indicators, which has the advantage of being simple and easy to determine, to access the relationship between elements and MetS components. Secondly, this is a preliminary exploration of the combined function effect of multiple elements on health outcomes. More professional statistical methods need to be further study and discussion. Thirdly, the information of dietary and physical activities factors were not available in this study, therefore the possibility of residual confounding of these factors could not be ruled out.

## Conclusions

There is indeed a association between the elements and the components of MetS in 6–12 years children. When different elements were analyzed individually and jointly, most of the conclusions were consistent, but some were different from single element analysis. Therefore, it is necessary to pay attention to the combined analysis of multi nutrients, which could provide new avenues for the early screening and prevention of chronic diseases. We will verify the relevant results through additional cohort research. and do more exploration in statistics to better analyze the combined effect of multiple elements in the future.

## Data Availability

The datasets used or analyzed during the current study are available from the corresponding author Lichen Yang on reasonable request.

## Abbreviations

MetS: Metabolic syndrome
AAP: American Academy of Pediatrics
IDF: International Diabetes Federation
BMI: Body mass index
TG: Triglycerides
HDL-C: High density lipoprotein cholesterol
FG: Fasting glucose
SBP: Systolic blood pressure
DBP: Diastolic blood pressure
